# Early Action Review of Detection, Notification, and Response Timeliness during Cross-Border Bundibugyo Virus Disease Outbreak, Uganda, 2026

**DOI:** 10.3201/eid3210.261411

**Published:** 2026-10

**Authors:** Joshua Kayiwa, Mudarshiru Bbuye, Ritah Atuhaire, Doreen Nyakato, Mathew Tut, Bayad Nozad, Jovan Baryamujura, Bernard Lubwama, Solome Okware, Pamela Kajumba, Grace Akello, Bonny Kintu, Rebecca Kahunde, Dativa Aliddeki, Motuma Guyassa, Lydia Nakiire, Felix Ocom, Paul Mbaka, Peter Elyanu, Allan Muruta, Atek Kagirita, Charles Olaro

**Affiliations:** National Public Health Emergency Operations Centre, Ministry of Health, Kampala, Uganda (J. Kayiwa, M. Bbuye, R. Atuhaire, D. Nyakato, J. Baryamujura, B. Lubwama, P. Kajumba, G. Akello, F. Ocom, A. Kagirita); Africa Centres for Disease Control and Prevention, Addis Ababa, Ethiopia (M. Tut, D. Aliddeki); UK Health Security Agency, London, UK (B. Nozad, M. Guyassa); World Health Organization, Kampala (S. Okware); Department of Integrated Epidemiology, Surveillance and Public Health Emergencies, Ministry of Health, Kampala (B. Kintu, A. Muruta); National Health Laboratory and Diagnostic Services, Ministry of Health, Kampala (R. Kahunde); Baylor Foundation Uganda, Kampala (L. Nakiire, P. Elyanu); Ministry of Health, Kampala (P. Mbaka, C. Olaro)

**Keywords:** Ebola virus, Bundibugyo virus, Bundibugyo virus disease, viruses, 7-1-7 target, early action review, epidemic preparedness, cross-border outbreak response, transboundary event, Uganda

## Abstract

Bundibugyo virus disease (BVD), an Ebola virus species with no licensed vaccine or therapeutic, reemerged in May 2026 as a cross-border outbreak in Uganda and the Democratic Republic of the Congo. During a 2-day workshop, July 8–9, 2026, we conducted an early action review of the outbreak response using the 7-1-7 framework (7 days to detect, 1 day to notify, 7 days to complete early response actions) to assess timeliness and identify bottlenecks and enablers across 9 response pillars. Uganda declared its outbreak on May 15, 2026; by July 8, the country had recorded 20 confirmed cases (15 imported, 5 locally transmitted) and a case-fatality rate of 15%. Uganda met all 3 targets: detection in 6 days, notification in <1 day, and response completion in 2 days. Low clinical suspicion, cross-border data-sharing gaps, fragmented digital systems, and delayed community engagement were common bottlenecks; strong leadership and coordination structures were most cited enablers.

Bundibugyo virus disease (BVD) is a severe viral hemorrhagic fever caused by *Orthoebolavirus bundibugyoense*, a documented Ebola virus species. The virus was first identified during a 2007 outbreak in Bundibugyo District, western Uganda (*1*); that outbreak ultimately comprised 131 cases, including 56 laboratory-confirmed cases and a case-fatality rate (CFR) of 40% among confirmed cases (*2*–*4*). Bundibugyo virus subsequently caused a second confirmed outbreak, in the Democratic Republic of the Congo (DRC) in 2012, comprising 62 cases, including 36 laboratory-confirmed cases and a CFR of 33% among confirmed cases (*5*). Reported CFRs for BVD are generally lower than for Ebola caused by Zaire ebolavirus (*6*). Unlike other Ebola virus species, no licensed vaccine or approved specific therapeutic currently exists for BVD, although licensed Zaire ebolavirus vaccines show early evidence of partial cross-reactive immunogenicity, and candidate BVD-specific vaccines and therapeutics are undergoing World Health Organization (WHO)–led prioritization (*7*–*11*).

On May 15, 2026, both Uganda and the DRC declared separate outbreaks, a transboundary BVD event, from which cases were epidemiologically linked to Ituri Province, DRC (*12*). Uganda’s index case, a 59-year-old DRC national, sought care at a private hospital in Kampala on May 11, 2026, and died on May 14, 2026 (*13*). On May 17, 2026, WHO determined the event constituted a Public Health Emergency of International Concern under the International Health Regulations 2005 (*14*), and on May 18, 2026, the Africa Centres for Disease Control and Prevention declared the outbreak a Public Health Emergency of Continental Security (*15*). By July 8, 2026, Uganda had recorded 20 confirmed cases, of which 15 were directly imported from the DRC, underscoring the cross-border nature of the outbreak and the need for sustained joint surveillance and coordinated screening along the Uganda–DRC corridor.

The 7-1-7 target, defined as <7 days to detect a suspected outbreak, <1 day to notify relevant public health authorities, and <7 days to complete essential early response actions, was jointly developed by WHO and Resolve to Save Lives, a nongovernmental global public health organization (*16*). The 7-1-7 target has been adopted by dozens of countries and institutions worldwide as a standardized, real-world performance benchmark for outbreak detection and response (*16*,*17*). Retrospective, multicountry evidence indicates substantial variation in how consistently those targets are met in practice (*18*,*19*), and Uganda has since institutionalized 7-1-7 monitoring as part of its national outbreak coordination platform (*20*); subsequent applications have been documented across other outbreak types in the country (*21*). WHO recommends that an early action review (EAR), an agile, structured methodology for assessing timeliness and identifying bottlenecks and enablers using the 7-1-7 tool, be conducted for all outbreaks of public health significance (*22*). The EAR formally assesses the timeliness of detection, notification, and response against the 7-1-7 targets, while enabling countries to evaluate coordination effectiveness within and across response pillars, identify pillar-level bottlenecks and enablers, and document emerging good practices.

Uganda conducted its EAR ≈8 weeks into the BVD response, as both an operational necessity and a formal obligation under the International Health Regulations 2005 Monitoring and Evaluation Framework (*23*). The process further assisted with development of actionable, time-bound recommendations to strengthen the ongoing response and inform national preparedness and readiness planning, in line with Uganda’s National Action Plan for Health Security (NAPHS), 2024–2029. In this article, we present the process, results, and lessons learned from that review.

## Methods

### Study Design and Setting

We conducted a retrospective, multipillar programmatic review using WHO’s standardized EAR methodology and 7-1-7 assessment tool (*22*). Nine response pillars were scoped for review under Uganda’s incident management system (IMS) structure: coordination; surveillance; laboratory; case management; risk communication and community engagement (RCCE); water, sanitation, and hygiene (WASH); logistics; strategic information, research, and innovation (SIRI); and continuity of essential health services (CEHS). We convened a 2-day workshop during July 8–9, 2026, bringing together participants from the Uganda Ministry of Health (MoH) and implementing and development partners, as well as experts from WHO, Africa Centres for Disease Control and Prevention, and the UK Health Security Agency.

### The 7-1-7 Framework

The 7-1-7 tool defines 3 sequential intervals: detection, the interval from the date of disease emergence (here defined as the earliest date on which a confirmed BVD case crossed the border into Uganda, thereby introducing the risk) to the date the event was first recorded by any source, against a target of <7 days; notification, the interval from the date of detection to formal notification of the public health authority responsible for action, against a target of <1 day; and response, the interval from notification to completion of the last of 7 defined early response actions (investigation, epidemiologic and risk assessment, laboratory confirmation, case management and infection prevention and control (IPC) measures, public health countermeasures, risk communication, and establishment of a coordination mechanism) against a target of <7 days (*16*,*17*).

### Data Collection and Synthesis

A total of 74 participants—38 from MoH, 12 from development, and 24 implementing partners—convened in 9 IMS pillar-based breakout groups, each supported by a trained facilitator and note-taker. Using the 7-1-7 standardized tool, each group reconstructed its pillar’s timeliness of detection, notification, and response actions; calculated interval timeliness against target; identified bottlenecks and enablers; and proposed immediate and longer-term remedial actions. On the second day, pillar findings were presented and discussed in plenary, enabling cross-pillar validation, consensus-building, and refinement of the consolidated bottlenecks, enablers, and recommendations. We subsequently consolidated findings centrally across all 9 pillars, and tagged each item to its source pillar for traceability.

### Ethics Considerations

This review constituted a programmatic evaluation of routine public health outbreak response activities using non–patient-identifiable process data. It was conducted under the MoH’s standing mandate for outbreak response and quality improvement and did not constitute human subjects research requiring separate ethical review.

## Results

### Outbreak Overview

By July 8, 2026, Uganda had confirmed 20 BVD cases: 15 imported from the DRC and 5 arising from secondary transmission within the country. The CFR stood at 15% among confirmed cases. Of the 831 contacts identified by then, 6 seroconverted, 4 died of causes unrelated to BVD, 1 was repatriated to DRC on request, and the remainder completed the standard 21-day follow-up according to existing guidelines. The country had tested >2,337 persons from 106 districts who were suspected of having any viral hemorrhagic fever (VHF), including samples from mortality surveillance. Case accrual slowed markedly as the response advanced. Uganda’s confirmed case count rose only marginally, from 19 cases as of June 17, 2026, to 20 by July 8, 2026, consistent with effective containment of secondary transmission following the initial response.

### 7-1-7 Timeliness

We summarized interval timeliness of the BVD response against the 7-1-7 targets (Table 1). Uganda’s earliest cross-border BVD case was traced to May 8, 2026 (date of emergence). The event was first recorded by the health system on May 14, 2026, when a community leader alerted the Director General of Health Services, giving a detection interval of 6 days (target <7 days). Formal notification occurred the same day, a notification interval of <1 day (target <1). The last of the 7 early response actions was completed on May 16, 2026, two days after notification (target <7). Uganda therefore met all 3 of the 7-1-7 targets for this event.

**Table 1:** Interval timeliness for World Health Organization 7-1-7 response target during cross-border Bundibugyo virus disease outbreak, Uganda, 2026*

Interval	Definition (dates)	Timeliness	Target	Target met
Detection	Emergence (May 8) → detection (May 14)	6 d	<7 d	Y
Notification	Detection (May 14) → notification (May 14)	<1 d	<1 d	Y
Response	Notification (May 14) → completion of last early response action (May 16)	2 d	<7 d	Y

*7-1-7, <7 days to detect a suspected outbreak, <1 day to notify relevant public health authorities, and <7 days to complete essential early response actions.

### Enablers by Pillar

We consolidated the enablers identified across all 9 pillar breakout groups, organized by pillar (Table 2). Three cross-pillar enablers were consistently cited: presence of strong senior leadership, reinforced by existing coordination architecture such as the National Task Force, the IMS, and the National Public Health Emergency Operations Centre, underpinning rapid activation across nearly every pillar; Uganda’s past experience in managing similar outbreaks, coupled with existing digital systems; and a trained, well-experienced, and ready-to-deploy workforce.

**Table 2:** Enablers of 7-1-7 timeliness during cross-border Bundibugyo virus disease outbreak, Uganda, 2026*

Response pillars	Actions
Coordination	
Detection	Proactive engagement from senior MoH leadership (Director General of Health Services) ensured rapid triage and verification of the initial signal, accelerating response actions.
Notification	The MoH officially declared the outbreak on May 15, 2026.
Response	Operationalization of the existing multihazard preparedness and response framework, which activated the National Task Force and IMS, enabling appointment of the Incident Commander by the DGHS within 3 h of confirmation.
	Presence of the National Public Health Emergency Operations Centre facilitated initial IMS activation and coordination mechanisms, including strategic, partner, pillar-specific, and daily IMS meetings.
Surveillance	
Detection	Community vigilance and involvement in surveillance enabled quicker identification and reporting of suspected cases.
Notification	A Kampala-based community leader from DRC was cooperative in reporting unusual health events, enabling early recognition and escalation of the initial signals.
Laboratory	
Response	Trained laboratory staff and adequate supplies and reagents for PCR testing and genomic sequencing enabled timely identification of BVD and correlation of the index case with the ongoing DRC outbreak.
	An efficient specimen referral hub network facilitated smooth transportation of suspected-outbreak samples to reference testing laboratories.
	Activation of district and regional laboratory pillar coordination for sample collection, transport, and reporting.
Case management	
Response	A well-equipped, established 84-bed ETU at Mulago National Referral Hospital plus a pool of ready-to-deploy trained staff supported swift admission and care.
RCCE	
Response	Availability of a central repository of English and translated Information Education and Communication materials, EVD-tailored messages, and guidelines for the public.
	Existing community structures including village health teams, community development officers, parish chiefs, and cultural and local council leaders were used for swift social mobilization and community-based surveillance.
	Strong collaboration between the MoH and the media network enabled timely dissemination of accurate messages.
WASH	
Response	Existing service contract frameworks for waste collection and management between the MoH and private service providers operating in the mapped high-risk districts.
	Inclusion of emergency personal protective equipment as a requirement in preexisting framework contracts/emergency procurement procedures.
	Adoption of a clustered deployment strategy at regional level to optimize team coverage.
Logistics	
Response	Pre-positioned emergency commodities at national medical stores, Mulago ETU, and Entebbe Isolation Unit, which were redistributed to the subnational level for immediate patient care.
SIRI	
Response	Availability of preprocured and functional ICT equipment, including internet and tablets for immediate deployment.
Cross-cutting	
Response	Readily available, competent national and subnational multidisciplinary rapid response teams deployed to high-risk districts.
	Previous national EVD response plans facilitated swift compilation of the current BVD response plan, accelerating prompt mobilization of resources by government and partners and operationalizing initial response mechanisms.
	Availability of existing and functional digital systems and dashboards, including ePHEM (https://pheoc.health.go.ug), eIDSR (https://eidsr.health.go.ug), Go.Data (https://vhf.health.go.ug), IRRDS (https://irrds.cphl.go.ug), the Uganda Health Alert System (https://alerts.health.go.ug), RESTRACK (https://restrack.cphl.go.ug), LIMS (https://rds.cphluganda.org), Action Tracker (https://actiontracker.health.go.ug), the Partner 4W matrix (https://partners.health.go.ug), and IORS (https://dashboards.health.go.ug), among others, which supported prompt data capture, analysis, and presentation, enabling rapid decision-making.

*7-1-7, <7 days to detect a suspected outbreak, <1 day to notify relevant public health authorities, and <7 days to complete essential early response actions. BVD, Bundibugyo virus disease; DGHS, Director General of Health Services; DRC, Democratic Republic of the Congo; ETU, Ebola treatment unit; EVD, Ebola virus disease; ICT, information and communication technologies; IMS, incident management system; MoH, Ministry of Health; RCCE, risk communication and community engagement; SIRI, strategic information, research, and innovation; WASH, Water, Sanitation and Hygiene.

### Bottlenecks and Action Matrix–Proposed Immediate and Longer-Term Recommendations

We compiled a list of bottlenecks identified for each pillar (Table 3). Two major cross-cutting patterns emerged. First, a low index of clinical suspicion, attributable to the nonclassical, symptom-predominant manifestations of the BVD strain rather than the classic hemorrhagic symptoms (*24*), recurred as a root cause in both the surveillance and case management pillars. Those bottlenecks contributed to delayed recognition, referral, and IPC uptake, especially in private healthcare facilities. Second, fragmentation of digital and information systems recurred across the surveillance, logistics, and SIRI pillars, manifesting as parallel electronic information systems, limited real-time stock visibility, and poor interoperability between reporting platforms. 

**Table 3:** Bottlenecks and actions by pillar from early action review of detection, notification, and response timeliness during cross-border Bundibugyo virus disease outbreak, Uganda, 2026*

Pillar and bottlenecks	Immediate action	Timeline	Long-term action
Pillar: Coordination			
Response: implementation of a few response activities outside pillar-specific structures	Formalize partner activity reporting through pillar coordination structures	Immediate	
Response: limited integration of mental health and psychosocial services within the response structure, contributing to initial responder fatigue and burnout	Provide an occupational health and welfare package for all responders	Immediate and continuous	
Pillar: Surveillance			
Detection: Vuura PoE lacked routine screening beyond 6 PM	Install prefabricated screening units at all gazetted PoEs to enable and sustain continuous screening of travelers regardless of travel time	Immediate	Fast-track approval/designation of permanent PoE human resources; construct permanent PoE infrastructure
Response: mortality surveillance not routinely implemented under nonresponse settings	Disseminate mortality surveillance guidelines, including standardizing reporting and digitalizing routine data tools	Immediate and continuous	Enhance training for mortality surveillance for all healthcare workers and disseminate appropriate data tools
Detection: late escalation of the initial community signal by a community leader Member of Parliament, which slightly delayed signal verification and investigation	Continued engagement with community leaders, including parliamentarians, on the basic principles of community-based surveillance	Immediate and continuous	Continued sensitization of community leaders in the basic principles of community-based surveillance
Detection: health workers had a low index of suspicion because cases had nonclassical symptoms of EVD	Conduct refresher training for health workers to strengthen clinical suspicion and reinforce surveillance and response protocols	Immediate and continuous	
Detection: limited cross-border surveillance information-sharing between DRC and Uganda	Periodic engagement with DRC surveillance teams to promote transparency in sharing cross-border surveillance information, especially for the current BVD outbreak and other infectious diseases	Continuous	Operationalize the existing signed MoUs to institutionalize routine cross-border collaborations, including surveillance zones, joint investigations and response simulation exercises, and quarterly PopCAB meetings.
Pillar: Laboratory			
Response: suboptimal use of e-LIS, driven by an initial lack of user accounts and inadequate training	Use RRTs at national and subnational levels for on-site and virtual e-LIS training; fast-track the ICT pathway to activate missing user accounts within 48 h of activation	Immediate	
Pillar: Case Management			
Detection: low index of suspicion among healthcare workers, leading to insufficient use of IPC measures and delayed notification or referral in private facilities	Massive distribution of job aids with clear case definitions and triage and screening protocols.	Immediate and continuous	Continuous medical education sessions at healthcare facility level to include revised case definitions and screening protocols
Response: unavailability of functional isolation and holding spaces in most private facilities	Engage private facilities to allocate isolation and holding spaces within their premises	Immediate and continuous	Strengthen subnational decentralization of functional isolation and treatment units in all RRHs
Pillar: Risk Communication and Community Engagement			
Response: suboptimal community engagement during the early phases of the response, which led to limited uptake of response interventions	Joint deployment of Risk Communication and Community Engagement teams	Immediate and continuous	
Response: limited funding for initial IEC material printing and scale-up	Leverage partnership with the private sector for IEC material printing and distribution		
Pillar: WASH			
Response: low literacy levels among some waste handlers, limiting efficient WASH uptake	Conduct refresher training for all waste handlers on effective WASH principles	Immediate and continuous	
Pillar: Logistics			
Response: dependence on central emergency stock acquisition and mobilization, which contributed to initial delays in emergency procurement activation	Maintain minimum emergency stock levels for EVD and other viral hemorrhagic fevers at national and regional PHEOCs/RRHs	Immediate and continuous	Institutionalize an emergency supply-chain activation protocol with expedited, digitized approval procedures
Response: suboptimal real-time tracking of outbreak commodities and supplies at district and regional levels	Quarterly inventory checks of emergency commodities at the subnational level	Quarterly	
Response: parallel commodity mobilization and incomplete logistics visibility, mostly fueled by infrastructure and utility failures	Establish an institutionalized, consolidated national logistics stock, pipeline, and distribution tracker integrating government and partner commodities	Continuous	Formalize logistics coordination and reporting requirements for all response pillars during IMS activation; strengthen ETU infrastructure resilience through preventive maintenance of utilities, oxygen systems, generators, and isolation facilities
Pillar: Strategic Information, Research and Innovation			
Response: limited interoperability among the multiple pillar-specific reporting systems	Strengthen interoperability and orient all pillars on approved response systems; ensure reporting compliance	Immediate and continuous	
Response: occasional server downtime and unavailability of online data-capture systems, which delayed some response reporting mechanisms	Strengthen server stability; ensure a backup internet solution; institutionalize URL whitelisting for all outbreak systems	Immediate and continuous	Install a backup internet ISP and functional power backup at the MoH data center

*BVD Bundibugyo virus disease; DRC, Democratic Republic of the Congo; e-LIS, electronic laboratory information system; ETU, Ebola treatment unit; EVD, Ebola virus disease; ICT, information and communication technologies; IEC, information, education, and communication; IMS, incident management system; ISP, internet service provider; MoH, Ministry of Health; MoU, memorandum of understanding; PHEOC, Public Health Emergency Operations Centre; PoE, point of entry; PopCAB, Population Connectivty Across Borders; RRH, regional referral hospital; RRT, rapid response team; URL, Uniform Resource Locator; WASH, Water, Sanitation and Hygiene.

For each bottleneck identified during the review, the corresponding pillar group proposed a remedial action, distinguishing actions for immediate implementation from actions requiring longer-term planning and funding, for example, through the NAPHS (2024–2029) or partner-funded programs (Table 3). All actions were validated in plenary on the second day of the workshop and discussed critically for feasibility and ownership by the respective pillar group.

## Discussion

This EAR found that Uganda met all 3 of the 7-1-7 targets during the 2026 cross-border BVD outbreak: detection achieved within 6 days, notification within 1 day, and completion of early response actions within 2 days. Meeting all 3 of the 7-1-7 targets simultaneously is an excellent finding in practice; a 5-country retrospective assessment of 41 public health events found that only 54% of events met the detection targets, 71% the notification targets, and 49% the response targets (*18*). Our results are thus consistent with Uganda’s early gains reported from its national 7-1-7 rollout (*20*,*21*). Similar EARs conducted for a cholera outbreak in Kenya (N. Roosevelt et al., unpub. data, https://doi.org/10.1101/2025.07.29.25332335) and a measles outbreak in Sierra Leone (*25*) have also identified actionable, pillar-specific bottlenecks, suggesting that the EAR methodology yields consistently useful operational insight across pathogens and settings. The low proportion of cases attributable to secondary transmission (5 of 20) further suggests that timeliness translated into a real reduction in onward transmission of a pathogen for which no licensed vaccine or therapeutic currently exists (*7*).

Despite the strong aggregate timeliness of the early BVD response, the review identified concrete opportunities for improvement. Generally, the ongoing transboundary BVD outbreak has illustrated the persistent clinical and public health challenges posed by filovirus disease outbreaks in resource-limited settings, including diagnostic delay and the demands of a coordinated field response (*26*). The recurrence of low clinical suspicion across both the surveillance and case management pillars is a notable finding; the clinical manifestation of cases in this outbreak was characterized predominantly by fever and gastrointestinal symptoms, rather than overt hemorrhage (*24*). Therefore, frontline health workers trained on a classic hemorrhagic case definition might have underrecognized the disease clinically, delaying isolation, IPC measures, and referral, particularly in private healthcare facilities. That initial gap in suspicion was rapidly narrowed by public awareness campaigns, intensified contact tracing, mortality surveillance, and point of entry (PoE) screening. We attribute the limited secondary transmission (5 of 20 cases) chiefly to those fast-follow interventions, rather than to missed cases during the outbreak’s earliest, low-suspicion phase. That finding further suggests that case definition training and job aids should explicitly incorporate the nonhemorrhagic manifestation of BVD as documented in this outbreak, rather than relying solely on generic Ebola virus disease case definitions. Similarly, the recurrence of digital and information-system fragmentation across the surveillance, laboratory, logistics, and SIRI pillars, including suboptimal electronic laboratory information system use, limited stock visibility, and poor system interoperability, points to a structural rather than pillar-specific weakness in Uganda’s outbreak information architecture. Such shortfalls are likely to recur in future events unless they are addressed at the systems level, rather than pillar by pillar.

The cross-border nature of this outbreak also foregrounded the need for real-time information-sharing with the DRC. Limited cross-border surveillance data-sharing was cited as a bottleneck despite the outbreak’s clear epidemiologic linkage to the parallel event in Ituri Province, DRC (*12*). That finding is further underscored by the fact that 15 of Uganda’s 20 confirmed cases were directly imported from the DRC, principally among persons in search of specialized medical care. Strengthening routine institutionalized cross-border collaboration, joint investigations, harmonized PoE screening, and regular bilateral coordination meetings were accordingly among the most frequently proposed immediate actions (Table 3), consistent with prior calls for stronger DRC–Uganda border health cooperation, given the recurring risk of spillover along this corridor (*19*). Such bidirectional dependence also means a well-prepared country can strengthen its neighbor’s early warning. Uganda’s own detection benefited from informal advanced notice of suspected Ituri Province cases, and the country has since extended preparedness support to healthcare facilities along the shared corridor.

The strong response-interval performance we identified also reflects a third cross-cutting pattern (Table 2): decisive senior leadership operating through existing coordination architecture. An incident commander was appointed within 3 hours of the initial case confirmation, and the National Task Force, IMS, and National Public Health Emergency Operations Centre were activated immediately, consistent with WHO’s requirement that a public health emergency operations center be fully activated within 120 minutes of detecting any public health event (*27*). The MoH was also able to make a public declaration of the event within the same day that the laboratory confirmed the index case. Of note, however, the coordination pillar’s own submission also reported a bottleneck of the opposite character: continued partner implementation of some response activities outside pillar coordination structures, leading to inconsistent response coordination. That finding indicates that strong central leadership did not, by itself, guarantee full coordination of all actors at the response start phase and that institutionalizing the coordination structures themselves, rather than relying on the persons who led this particular response, warrants continued attention (*28*).

Many of the enablers we identified reflect capacities built over nearly 2 decades of response to recurrent filovirus outbreaks in Uganda, including the 2007 BDV event (*2*–*4*). Those events catalyzed sustained investment in ready-to-deploy rapid response teams, national and regional public health emergency operations centers, mobile laboratory capacity, prepositioned emergency stocks, and predeveloped risk communication materials, among other capacities. Countries lacking such infrastructure might need to apply a narrower subset of the enablers to achieve comparable 7-1-7 performance.

Beyond the specific bottlenecks discussed above, the pillar-generated action matrix (Table 3) warrants critical comment on implementation feasibility. Surveillance alone accounts for 5 of the 17 immediate actions, raising a practical question about parallel implementation capacity within an already-stretched pillar team. Second, several longer-term actions, such as the proposed salary approval for permanent PoE staffing through the NAPHS (2024–2029), depend on external budget cycles beyond MoH’s direct control. Third, although digital and information-system fragmentation was identified as a single, cross-cutting problem, the proposed actions remain pillar-siloed (e.g., electronic laboratory information system training, a logistics tracker, and SIRI interoperability) rather than converging on 1 cross-pillar strategy that Uganda’s post-EAR action tracker should pursue. Finally, several of the identified bottlenecks, including inconsistent mortality surveillance, limited community leader familiarity with community-based surveillance, and constrained cross-border data-sharing, have also recurred across other unpublished Uganda-specific EARs, reflecting structural rather than outbreak-specific weaknesses that warrant systemwide remediation.

The first limitation of this review is that it was conducted during the eighth week after outbreak declaration rather than within the recommended first or second week (*22*), limiting the extent to which findings could inform real-time course correction of the still-active readiness and response efforts. That delay was deliberate; convening the same response staff in a 2-day workshop earlier, while case counts were still rising, risked distracting from active outbreak control, a tradeoff likely more acute in settings with larger or still-escalating caseloads, such as the concurrent DRC response. Second, although the health system director and other senior leaders credited as enablers did not personally attend EAR discussions, social-desirability bias among participants cannot be excluded; anonymized feedback channels could mitigate that effect in future EARs. Third, the CEHS and WASH pillar actions were documented within other pillars’ submissions, including surveillance and logistics, explaining why some of their input is not separately visible (Table 3). That limitation further reflects the interlinked, complementary nature of response activities and that no single pillar can mount a response action in isolation from others (*29*). Finally, as a single-country case study of an outbreak consisting of predominantly imported cases, findings on absolute timeliness might not generalize to settings with different surveillance infrastructure or to outbreaks driven primarily by endemic in-country transmission. However, the recurring bottleneck themes, including clinical suspicion, information-system fragmentation, and cross-border data-sharing, could plausibly extend to other Ebola-endemic contexts within the Great Lakes region of Africa.

In conclusion, this EAR found that Uganda met all 3 of the 7-1-7 targets during the 2026 cross-border BVD outbreak: detection in 6 days, notification in <1 day, and completion of early response actions in 2 days. That result outperforms published multicountry benchmarks and was associated with limited secondary transmission. Those gains reflect the value of proactive leadership and coordination architecture in this response, backed by capacities built over nearly 2 decades of prior filovirus responses. The review further identified actionable, pillar-specific opportunities: reinforcing clinical suspicion for atypical disease manifestations, institutionalizing cross-border surveillance collaboration with the DRC, integrating fragmented digital and laboratory information systems, and accelerating community engagement. Incorporating those recommendations into Uganda’s NAPHS (2024–2029), and conducting future EARs earlier in an outbreak course where feasible, can further strengthen national capacity to detect, notify, and respond to high-consequence pathogens.
